# A One-Step Miniprep for the Isolation of Plasmid DNA and Lambda Phage Particles

**DOI:** 10.1371/journal.pone.0023457

**Published:** 2011-08-15

**Authors:** George Lezin, Yasuhiro Kosaka, H. Joseph Yost, Michael R. Kuehn, Luca Brunelli

**Affiliations:** 1 Division of Neonatology, Department of Pediatrics, The University of Utah School of Medicine, Salt Lake City, Utah, United States of America; 2 Department of Neurobiology and Anatomy, The University of Utah School of Medicine, Salt Lake City, Utah, United States of America; 3 Laboratory of Protein Dynamics and Signaling, National Cancer Institute, National Institutes of Health, NCI-Frederick, Frederick, Maryland, United States of America; University of Massachusetts Medical School, United States of America

## Abstract

Plasmid DNA minipreps are fundamental techniques in molecular biology. Current plasmid DNA minipreps use alkali and the anionic detergent SDS in a three-solution format. In addition, alkali minipreps usually require additional column-based purification steps and cannot isolate other extra-chromosomal elements, such as bacteriophages. Non-ionic detergents (NIDs) have been used occasionally as components of multiple-solution plasmid DNA minipreps, but a one-step approach has not been developed. Here, we have established a one-tube, one-solution NID plasmid DNA miniprep, and we show that this approach also isolates bacteriophage lambda particles. NID minipreps are more time-efficient than alkali minipreps, and NID plasmid DNA performs better than alkali DNA in many downstream applications. In fact, NID crude lysate DNA is sufficiently pure to be used in digestion and sequencing reactions. Microscopic analysis showed that the NID procedure fragments *E.coli* cells into small protoplast-like components, which may, at least in part, explain the effectiveness of this approach. This work demonstrates that one-step NID minipreps are a robust method to generate high quality plasmid DNA, and NID approaches can also isolate bacteriophage lambda particles, outperforming current standard alkali-based minipreps.

## Introduction

Plasmid DNA minipreps are essential for molecular biology research and rely on disrupting *Escherichia coli* (*E. coli*) cell walls. These procedures can be classified according to the disruptive factor used. Alkali procedures use NaOH with SDS [1], as well as NaOH with either zwitterionic [2], or non-ionic detergents (NIDs) [3]. Detergent procedures use SDS alone [4], or in combination with pronase [5] or lysozyme [6]. Organic extraction procedures use either phenol or phenol/chloroform mixtures alone [7], [8], [9], [10], [11], or in combination with Triton X-100 [12], or lysozyme [13], [14]. Physical treatment procedures include boiling alone [15], or boiling in solutions of lysozyme with either Triton X-100 [16] or Tween 20 [17]. Finally, following the attempts to isolate polysomes without gross physical disruption of *E. coli* cells using lysozyme and the NID Brij-58 [18], a procedure for plasmid DNA isolation was developed by adding 0.2% sodium deoxycholate (DOX) to Brij-58-containing lysis buffers [19]. A further development of this approach used Brij-58 and DOX in highly hypertonic salt-sucrose solutions [20]. Among the procedures developed over the last 40 years, alkali isolation is currently the most widely used plasmid DNA miniprep [1], and provides the basis for most kit-based minipreps on the market. This approach is fairly reliable, but requires some experience to prevent contamination of plasmid DNA with genomic DNA, and bacterial cells require incubation with three different solutions. Plasmid DNA also usually requires subsequent column purification steps, probably because of SDS contamination. Finally, alkali-based approaches can only extract circular DNA, and not other bacterial extra-chromosomal elements, such as bacteriophages, which can be used as vectors for the construction of genomic and cDNA libraries [21].

NID-based plasmid DNA isolation procedures have been used in a multiple-solution format, but they have not gained significant popularity [3], [19], [20]. NIDs are soluble amphipathic molecules consisting of polar (hydrophilic) and nonpolar (hydrophobic) moieties which can solubilize membrane lipids [22]. The hydrophile-lipophile balance (HLB) determines their water solubility [23], [24], where a lower HLB value indicates that a detergent is less hydrophilic. We have recently shown that NIDs outperform column-based methods in plasmid DNA purification procedures [25], but it remains unclear whether NIDs may also be used to develop a one-step plasmid DNA isolation procedure.

Here, we describe a robust and time-efficient procedure using NIDs that provides a one-tube, one-solution approach to isolate plasmid DNA. Interestingly, a slightly modified NID procedure also isolates bacteriophage lambda particles. Unlike the complete cell disintegration occurring in alkali minipreps, NID plasmid minipreps lead to fragmentation of *E.coli* cells into small protoplast-like components.

## Materials and Methods

NIDs, salts and lysozyme were purchased from Sigma–Aldrich (St. Louis, MO) or Mallinckrodt Baker (Phillipsburg, NJ). RNase A was purchased from 5 PRIME (Gaithersburg, MD). Antibiotics were purchased from USB (Cleveland, Ohio), Sigma–Aldrich, and EMD/Calbiochem (Gibbstown, NJ). Bacterial media were obtained from Quality Biological (Gaithersburg, MD). DH5α cells were supplied by Invitrogen (Carlsbad, CA). The following high copy number plasmids were used: 1) pUC19 (2.7 Kb), purchased from Invitrogen (Carlsbad, CA); 2) pCYPAC3 (18.8 Kb) [26], a pUC-based plasmid, kindly provided by S. O′Brien (NCI, Frederick, MD); 3) pLTM330 (6.5 Kb), a pBluescript-based plasmid, kindly provided by L. Tessarollo (NCI, Frederick, MD); and 4) B254 (6.06 Kb), a pBluescript-based plasmid, kindly provided by E. Leibold (University of Utah, Salt Lake City, UT). pEL04 (5.07 Kb, ts pSC101 oriR), a low copy number plasmid (Qiagen® Plasmid Purification Handbook 3^rd^ Edition, Nov 2005, pg. 12), was kindly provided by NCI-Frederick, MD (http://web.ncifcrf.gov/research/brb/ productDataSheets/recombineering/plasmid.aspx). *E.coli* cells UB-61 lysogenic for a heat-inducible bacteriophage lambda (*cI857 ind 1 Sam7*) was kindly provided by S. Casjens (University of Utah). All enzymes were purchased from New England Biolabs (Ipswich, MA), except PstI (Promega), and SstI and XhoI (Invitrogen). Enzymatic reactions were performed at 37°C for 1 hour if not otherwise specified.

### DNA quantification

DNA was quantified using a two wavelength spectrophotometric method on nanodrop spectrophotometer (Thermo Fisher Scientific, Waltham, MA).

### Standard alkali DNA isolation

Alkali isolation was performed according to the original description by Birnboim and Doly [1].

### NID miniprep plasmid isolation procedure

1. 1.5-2 ml of bacterial cultures were pelleted at 6000-7000 rpm for 1 min.
2. After drawing 150 µl extraction buffer into a pipette tip, the pellet was loosened off the tube wall with the tip without releasing the buffer. Then the extraction buffer was added and the pellet resuspended.
3. The bacterial suspension was incubated at 65°C for 5 min.
4. Suspensions were centrifuged at maximum rpm for 10 min or until a tight bacterial pellet was formed. The pellet was removed with a toothpick.
5. 100-120 µl isopropanol was added, followed by mixing and centrifugation of the solution at 7000 rpm for 10 min at RT.
6. DNA usually forms film-like precipitates that adhere well to tube walls and are invisible in isopropanol solutions. After discarding the supernatant, the DNA was centrifuged after adding 70% ethanol. Ethanol was removed, and the DNA pellet was dissolved in 20-50 µl TE buffer.

The composition of the extraction buffer was: 5% sucrose, 20–50 mM EDTA, 50 mM Tris pH 8, 0.75 M NH_4_Cl, 0.5% IGEPAL CA-630 (or Triton X-100), lysozyme 100 µg/ml, and RNase A 25 µg/ml. Addition of 20–50 mM CaCl_2_ to the extraction buffer reduces extraction of chromosomal DNA and large plasmids, but greatly facilitates formation of cellular debris during sedimentation. A 100x enzyme stock containing 10 mg/ml lysozyme and 2.5 mg/ml of RNase A prepared in 50% glycerol and 50 mM Tris pH 8 was stored at −20°C and used repeatedly.

### Densitometry

Image densitometry/gel quantification analysis was performed using Image J [27]. All gel images were calibrated in OD units using Kodak No. 3 Calibrated Step Tablet.

### DNA sequencing

DNA sequencing was performed using the ABI BigDye Terminator Cycle Sequencing Kit v1.1 (NID DNA) or v3.1 (NID crude lysates) according to the manufacturer's instructions on a Gene Amp 9700 PCR machine. The primers used were M13f (GTA AAA CGA CGG CCA GT) for sequencing B256 plasmid, and r pLTM330_3617 (GCT GGT TCT TTC CGC CTC A). The sequence fragments were detected on an ABI 3130XL Genetic Analyzer. Samples were then analyzed and base-called by Applied Biosystems DNA Sequencing Analysis Software V5.2 (Applied Biosystems, Foster City, CA).

### Mathematical model

To estimate time-efficiency of the NID relative to the alkali procedure, the total NID and alkali procedure completion times (T_tot_) were considered as the sum of the operational time (time dependent on the number of samples, T_op_) and the preparation/working equipment times (time independent on the number of samples, time idle, T_id_). T_tot_ =  T_op_ + T_id_. T_op_ is a product of *k* (a constant, average time for isolating 1 sample) by n (number of samples). T_op_ =  *k*n. Thus, T_tot_ =  *k*n + T_id_ where T_id_ is a constant (*c*). T_tot_/T_op_ =  1 + *c*/*k*n, showing that as the number of samples increases T_tot_/T_op_ approaches 1. In other words, as n increases, T_tot_ and T_op_ can be used interchangeably.

*k* and the standard error were estimated by linear regression analysis (T_op_ as a function of n) using the data presented in Table 1, forcing the fitting line to intersect the point of origin. Microsoft Excel LINEST function was used, with const is FALSE and stats is TRUE.

**Table 1:** Time requirements of alkali vs. NID plasmid minipreps.

Number of preps	Total time, min		Operator-dependent time, min		Ratio of operator-dependent to total time, %	
	A	N	A	N	A	N
1	26	30	6	5	23	17
5	35	35	15	10	43	29
10	42	40	22	15	52	37
15	53	44	33	19	62	43
20	62	49	42	24	68	49
24	66	54	46	29	70	54
30	78	59	58	34	74	58

Both procedures were performed without rinsing DNA pellets with 70% ethanol. Distriman repetitive pipette (Gilson, Inc., Middleton, WI) was used to dispense solutions. Initiation of next step of procedure was not allowed until ongoing step was completed. The total procedure time was the sum of operational time and the preparation/working equipment times, such as incubators and centrifuges (see Materials and Methods).

### Bacteriophage lambda particle isolation

Lysogenic *E.coli* UB-61 cells were grown at 30°C until OD_600_ =  1. The culture was divided into 12–15 ml aliquots and heat-shocked for 10 min at 42–43°C, then re-combined and incubated at 37°C for 7–8 hours. One or two 5 ml aliquots were lysed with chloroform (full lysis sample). The other 5 ml aliquots were spun down and the cells were resuspended in the following lambda NID extraction buffer: 5% sucrose, 50 mM Tris pH 8, 2 M NH_4_Cl, 50 mM CaCl_2_, lysozyme 120 µg/ml and 0.5% Tween 80. No EDTA was included in this buffer. Bacteria were incubated at 40°C for 45 min with occasional mixing to prevent bacterial sedimentation, and centrifuged at 12–15,000 rpm for 5–10 min. Easy cell sedimentation and absence of viscous pellets suggested no lysis of bacteria. The supernatant was precipitated with 1∶1 volume 1.25 M NH_4_Cl and 20% PEG 8000 after 1 hour incubation in ice.

The full lysis samples were precipitated with the following solution: 1 M NaCl and 10% PEG 8000 added as powder.

The phage containing pellets were processed according to Sambrook et al. with minor modifications [21]. Shortly, they were gently resuspended in 0.2–0.5 ml modified SM buffer (25 mM Tris pH 7.6, 75 mM NaCl, 10 mM MgCl_2_) containing >5 Kunitz units/ml DNaseI and incubated at 37°C for 1 hour. Subsequently, 15 mM EDTA was added. DNA was extracted with phenol/chloroform and before isopropanol precipitation the aqueous phase was mixed with 20 µg linear polyacrylamide carrier to ensure quantitative DNA recovery [28]. After centrifugation supernatant was carefully aspirated and the pellets were dissolved in 40 µl TE containing 30 µg/ml RNase A.

### Plasmid DNA miniprep according to Godson and Sinsheimer using Brij-58 [18]

Bacterial cells from 2 ml cultures were resuspended in 60 µl Godson's sucrose solution (25% w/v sucrose in 10 mM Tris pH 8.1). Cells were kept on ice throughout the procedure. We mixed in advance 7.5 µl 16 mM EDTA and 7.5 µl 0.85 mg/ml lysozyme in 250 mM Tris 8.1. This solution was mixed with the bacterial suspension and incubated on ice for 1 min. The bacterial suspension was then transferred to a lytic mixture containing 15 µl 5% Brij-58 in 10 mM Tris pH 7.4 and 60 µl deionized water and incubated on ice for 10 min. We excluded 10 mM MgSO_4_ from the original lytic mixture and increased the last incubation time from 2 to 10 min. to increase the amount of plasmid DNA extracted. The mixture was then centrifuged at 5000 RPM (3000 ×g) for 5 min, and the cleared lysate was precipitated with 150 µl isopropanol. DNA pellets were resuspended in 35 µl TE buffer containing 30 µg/µl RNase A. The insoluble material was spun down and the cleared DNA solution was loaded on a gel.

### Plasmid DNA miniprep according to Clewell and Helinski using Brij-58 and DOX [19]

Bacterial cells from 2 ml cultures were resuspended in 47 µl Clewell's sucrose solution (25% sucrose in 0.05 M Tris, pH 8.0). Cells were kept on ice throughout the procedure. Subsequently, 10 µl 5 mg/ml lysozyme in 250 mM Tris pH 8 was added, and the solution was kept on ice for 5 min. 19 µl 0.25 M EDTA was added and incubation continued for another 5 min. 76 µl detergent mixture (1% Brij-58, 0.4% DOX, 62.5 mM EDTA, 50 mM Tris, pH 8.0) was then added to the 76 µl bacterial suspension and kept on ice for 5–10 min. The mixture was then centrifuged at 48,000g at 2°C for 25 min. The cleared lysate was precipitated with 150 µl isopropanol, and the DNA pellets were resuspended in 35 µl TE buffer containing 30 µg/µl RNase A. The insoluble material was spun down and the cleared DNA solution was loaded on a gel.

### Plasmid DNA miniprep according to Summerton et al. using Brij-58 and DOX in highly hypertonic salt-sucrose solutions [20]

Bacterial cells from 2 ml cultures were resuspended in 60 µl DEPC/Summerton's sucrose solution (1 µl DEPC/500 µl Summerton's sucrose solution: 100 mM Tris pH 8.1, 30% sucrose, 100 mM EDTA). The cells were incubated on ice for 5 min. Subsequently, 15 µl lysozyme (6 mg/ml in water) was added and incubation continued for 20 min in ice. An equal volume (75 µl) ice cold salt-detergent solution (0.4% DOX, 1% Brij-58, 2 M NaCl) was added, gently mixed and incubated at 25°C for 20 min without mixing. The mixture was then centrifuged at 40,000 g for 30 min at 0°C. The cleared lysate was precipitated with 150 µl isopropanol, and the DNA pellets were resuspended in 35 µl TE buffer containing 30 µg/µl RNase A. The insoluble material was spun down and the cleared DNA solution was loaded on a gel.

### Imaging of bacterial suspensions

20–40 µl bacterial cell lysates from the various isolation methods were placed on slides and mounted with cover slips. Images were recorded using either an Olympus IX81/Metamorph v6.2r6 microscope (100x objective in oil) or a Leica DMR A/Metamorph v6.3r1 (63x objective in oil).

## Results

### Effective plasmid DNA extraction using NIDs, osmolytes, and elevated temperatures

**Figure 1:**
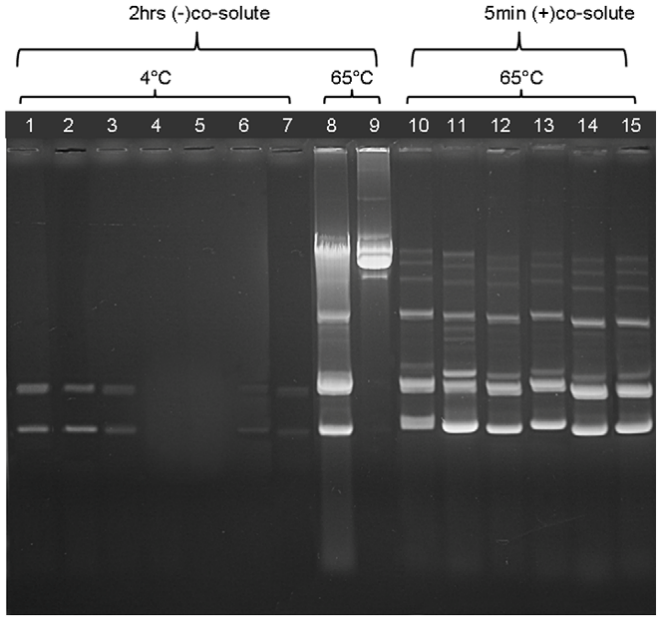
Elevated temperatures and osmolytes increase the efficiency of plasmid DNA extraction by NIDs. Extractions of either pUC19 (lanes 1–8 and 10–15) or pCYPAC3 plasmids (lane 9) were carried out. Transformed DH5α cells were grown in 1.5 ml LB cultures and resuspended in 150 µl 50 mM Tris pH 8, 10 mM EDTA with or without co-solutes. 500 µg/ml lysozyme was also added, and the cells were incubated as specified below. Salt concentrations were adjusted to 0.5 M NaCl in all extracts, except the ones loaded in lanes 10 and 12–15. Extracts were cleared by centrifugation, and precipitated with 150 µl isopropanol. DNA pellets were dissolved in 40 µl TE buffer, 40 µg/ml RNase A. 10 µl aliquots of the solutions containing either the pCYPAC3 (lane 9) or pUC19 plasmids (all other lanes) were loaded on the gel. Exposure times and temperatures of extraction are shown above the lanes. For lanes 1–9, extraction with the indicated NID was done in the absence of co-solutes. For lanes 10–15, the included co-solute is indicated. Lane 1: 0.5% IGEPAL CA-630. Lane 2: 0.5% TX-100. Lane 3: 0.5% IGEPAL CA-720. Lane 4: 0.5% Tween-80. Lane 5: 0.5% Tween-20. Lane 6: 2% IGEPAL CA-720. Lane 7: 4% IGEPAL CA-720. Lane 8: 0.5% Tween 80. Lane 9: 0.5% Tween 80. Lane 10: 0.5% Tween 20/0.5 M KCl. Lane 11: 0.5% Tween 20/22.5% sucrose. Lane 12: 0.5% IGEPAL CA-630/0.5 M NH_4_Cl. Lane 13: 0.5% TX-100/0.5 M NH_4_Cl. Lane 14: 0.5% TX-100/0.5 M NaCl. Lane 15: 0.5% TX-100/0.5 M NaAc.

To evaluate how different NIDs perform in plasmid DNA extraction, we used various NIDs mixed with lysozyme-treated DH5α cells harboring pUC19. Plasmid DNA was extracted effectively when *E.coli* were exposed to NIDs with HLBs <15 (IGEPAL CA-720, Triton X-100 and IGEPAL CA-630) for 2 hours at 4°C (Figure 1, lanes 1–3), but other NIDs, such as Tween 80 and Tween 20 were ineffective at these conditions (Figure 1, lanes 4–5). A 0.5% NID concentration was more effective for DNA extraction compared to increasing NID concentrations up to 4% (shown for IGEPAL CA-720, Figure 1, lanes 3, 6, 7). The efficiency of DNA extraction increased markedly at 65°C and was similar for all NIDs (shown here only for Tween 80, Figure 1, lane 8). Under these conditions, plasmid DNA up to 19 Kb (pCYPAC3) was also extracted well (Figure 1, lane 9). To decrease extraction time, we tested the effects of osmolytes. The addition of various osmolytes (sucrose, NaCl, KCl, NH_4_Cl and NaAc) to the NID solutions decreased the required NID extraction time down to 5 minutes for all NIDs studied (Figure 1, lanes 10–14). Other osmolytes tested (LiCl, LiAc, KAc, EDTA, glucose, NH_4_Ac and NH_4_HCO_3_) were effective but differed in their propensity to extract chromosomal DNA and precipitate DNA in aqueous isopropanol solutions (data not shown). These data demonstrate that osmolytes and elevated temperatures enhance the activities of a variety of NIDs, allowing rapid and efficient extraction of plasmid DNA in a single solution format.

### NID miniprep plasmid DNA is a robust substrate for digestion, ligation, and sequencing

**Figure 2:**
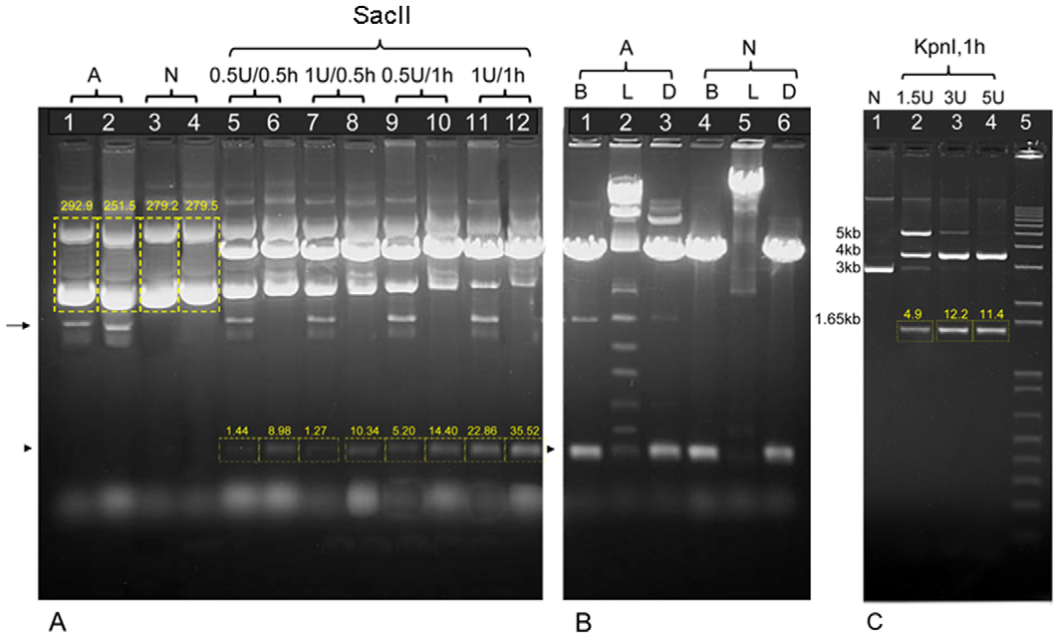
NID-extracted DNA of both high and low copy number plasmids perform well in common downstream applications. (**A**) Restriction endonuclease digestion of the high copy number pLTM330 plasmid containing 2 SacI sites separated by approximately 400 bp. extracted by either alkali or NIDs. XL1-Blue cells harboring pLTM330 in 1.5 ml LB cultures were used for isolation. After alcohol precipitation, alkali DNA pellets were rinsed with 0.5 ml ethanol and dissolved in 40 µl TE buffer, but NID DNA pellets were directly dissolved in 40 µl TE buffer. 9 µl aliquots of native DNA of two independently isolated samples were loaded in lanes 1, 2 (alkali, A) and lanes 3, 4 (NID, N). Densitometry analysis is reported for lanes 1–4. 9 µl DNA was also used for every DNA restriction and ligation reaction in 11 µl total volume. Restriction digests with the indicated amount of restriction enzyme for the indicated length of incubation at 37°C are shown in lanes 5, 7, 9, 11 for the alkali method and lanes 6, 8, 10, 12 for the NID protocol. Arrow indicates “irreversibly denatured” DNA [1], [25], [29]. Arrowhead indicates the 400 bp band, and densitometry analysis of this band is reported for lanes 5–12. DNA restriction reactions were carried out in NEB1 buffer. (**B**) Plasmid samples digested using 2u SacI for 1 hour (lanes 1,4) were ligated using 0.1 Weiss unit T4 DNA ligase at 15°C for 30 minutes (lanes 2,5). Ligation products were then re-digested using 2u SacI for 1 hour (lanes 3,6). DNA restriction and ligation reactions were carried out in NEB1 buffer, but 1 mM ATP was added for the ligation reactions. Arrowhead indicates 400 bp band. B =  cut sample. L =  ligated sample. D =  re-cut sample. Lane 2 shows the intermediate ligation products of the 400 bp DNA fragment in lane 1. (**C**) DH5α cells harboring the low copy number plasmid pEL04, containing 2 KpnI sites separated by approximately 1.5 Kb. NID plasmid isolation was performed as reported in Materials and Methods, except that lysozyme concentration in the extraction buffer was 50 µg/ml. 9 µl DNA in 11 µl total reaction volume were used for digestions. Lane 1: native DNA (N). Lane 2: DNA digestion by 1.5u KpnI. Lane 3: 3u KpnI. Lane 4: 5u KpnI. The incubation times were 1 hour. Lane 5: 1 Kb Plus DNA Ladder (Invitrogen). Densitometry analysis of the expected 1.5 Kb digestion product is reported in lanes 2–4.

We next compared performance of the pLTM330 plasmid DNA obtained by either NID or alkali miniprep in digestion, ligation and sequencing reactions. The NID and alkali minipreps produced similar amounts and molecular forms of DNA, except for some “irreversibly denatured” (fast migrating, single stranded) DNA forms in the alkali method (Figure 2A, lanes 1,2 vs. 3,4, arrow) [1], [25], [29]. The plasmid used for these experiments contains 2 SacI sites separated by approximately 400 bp, and appearance of this band was analyzed by densitometry. At two concentration (0.5 vs. 1u) and incubation times (0.5 vs. 1 hr), the endonuclease SacI digested NID miniprep DNA more efficiently than alkali miniprep DNA, as determined by densitometry quantification of the 400 bp band (Figure 2A, lanes 5–12, arrowhead). NID miniprep DNA also performed significantly better in ligation reactions. The major ligation product had a higher molecular weight and intermediate products were virtually absent compared to alkali isolation (Figure 2B, lanes 2, 5). Ligated NID DNA was completely re-cut using SacI (Figure 2B, lanes 3, 6).

Isolation of low copy number plasmids requires higher bacterial culture volumes, which might result in increased DNA impurities. Thus, we isolated pEL04, a low copy number plasmid, using the NID procedure and then assessed digestion. We isolated DNA from 2 ml cultures (vs. 1.5 ml in high copy number plasmids), and dissolved the DNA in 20 µl TE buffer (vs. 40 µl in high copy number plasmids). As the amount of DNA was enough for only two digestions, we combined DNA from three independent samples. We used the salt-sensitive restriction endonuclease KpnI, with expected digestion products of 1.5 and 3.5 Kb. We found that 3u KpnI are sufficient to achieve almost complete digestion (Figure 2C). These data show that the NID procedure can be used to isolate DNA of low copy number plasmids.

**Figure 3:**
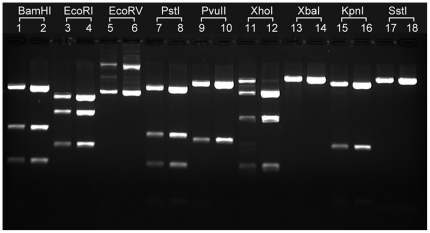
NID crude lysates perform well in digestion reactions. 2 ml bacterial cultures containing plasmid B254 were processed according to the NID miniprep plasmid isolation procedure with (complete procedure, CP) or without (crude lysate, CL) isopropanol precipitation. 50 mM MgSO_4_ was added to the CL to chelate 50 mM EDTA in the extraction buffer. All CL samples were digested in 15 µl NEB 1 buffer (no salt), and 1 µl CL was used for digestions. CP samples were digested in the specific NEB buffers recommended for the restriction endonuclease, and 0.5 µl DNA was used for every reaction. Samples were incubated with 5u of each enzyme at 37°C for 1 hr. The reaction products of CL digestion were loaded in odd-numbered lanes, while digestion products of CP DNA were loaded in even-numbered lanes.

Crude lysates of alkali minipreps cannot be used for downstream applications because of their low DNA concentration, SDS content and acidic pH. NID minipreps are free of these shortcomings, leading us to examine whether NID crude lysates could be directly used in downstream applications, such as digestion and sequencing reactions. We assessed how NID plasmid DNA from crude lysates (i.e., NID miniprep, steps 1–4) performed in digestion reactions compared to isopropanol precipitated-NID plasmid DNA (i.e., NID miniprep, steps 1–6). All tested restriction endonucleases except XhoI digested crude lysate DNA well (Figure 3). DNA molecular forms were similar when NID crude lysates were immediately precipitated with isopropanol or stored overnight at RT (data not shown). The possible effects of storage were tested because covalently closed circular (CCC) plasmid DNA derived from NID-based procedures can be relaxed by heat and/or storage in EDTA containing solutions [19], [30]. We also found that NID miniprep plasmid DNA is a robust template in sequencing reactions without requiring any additional purification. The read length was 800 nucleotides (nt) with 5 ambiguous nt in the first 39 nt (accession number JF804976). Interestingly, NID crude lysates could also be used in sequencing reactions because as little as 1 µl crude lysate generated reliable sequencing data with trimmed length 783 b (Figure S1A). Increasing the amount of crude lysate to 3 µl improved the trimmed length slightly to 804 b, but 4 µl crude lysates reduced it significantly to 523 b (Figure S1B, C). Taken together, these data provide evidence that NID minipreps can be reduced to a one-step procedure (i.e., isopropanol precipitation is not required). NID miniprep plasmid DNA is highly suited for a variety of common downstream applications, and outperform standard alkali miniprep DNA in digestion and ligation reactions, possibly because of higher purity.

### NID minipreps are time-efficient compared to the alkali miniprep

Alkali minipreps require three solutions for completion of plasmid DNA isolation, but NID minipreps require only one. To confirm that NID minipreps are more time-efficient than alkali minipreps, we compared completion time up to thirty samples of alkali and NID minipreps. The time required to complete one miniprep was similar, although the NID miniprep required relatively less operator-dependent time (Table 1). As the number of samples increased, the time advantage of the NID miniprep increased significantly for both the total and operator-dependent times. For thirty minipreps, the total time was 78 min. for alkali, but only 59 min. for NID minipreps, while the operator-dependent time was 58 min. for alkali vs. 34 min. for NIDs (Table 1). In addition, the ratio of the operator-dependent time to the total time was 74% with alkali vs. 58% with NID minipreps (Table 1).

Claiming a time advantage of a procedure over previous ones is often difficult because it is hard to evaluate the data and extrapolate the conclusions to any sample number. To address these issues, we developed a linear regression mathematical model. When the same operator completes both the alkali and NID procedure, the total procedures times are dependent variables as shown in Table 1 (linear correlation coefficient, r, 0.988, implying that T_alk_ =  *a*T_nid_+*b*, where *a* and *b* are constants). Thus, the ratio of the variables T_alk_/T_nid_ =  *a*+*b*/T_nid_ =  *a*+*b*/n*k* (see Materials and Methods). When the number of samples (n) is high, *b*/n*k*∼0, and T_alk_/T_nid_∼*a*, a constant. This suggests that although alkali and NID operational times can change based on the operator, their ratio tends to be invariant. Using the operator-dependent times in Table 1, *k* was calculated as 2.03±0.06 min. (coefficient of determination, r^2^ = 0.995) and 1.20±0.05 min. (r^2^ = 0.991) for the alkali and NID minipreps, respectively. Thus, at high sample numbers (when operator-dependent time can replace total time), NID minipreps are 69% more time-efficient compared to alkali minipreps (2.03/1.20 – 1×100). Overall, these data demonstrate that NID minipreps save significant time compared to alkali minipreps, and suggest that NID minipreps are particularly suitable for high-throughput applications.

### NID minipreps effectively isolate bacteriophage lambda particles

**Figure 4:**
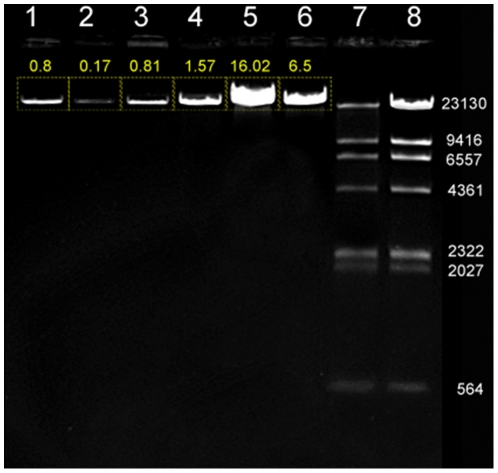
Isolation of lambda phage DNA using NID-based extraction. The DNA samples loaded in lanes 1–5 were generated varying only the composition of the phage particle extraction buffer. The particles were precipitated and the lambda DNA was extracted from all samples using the same procedure as described in Materials and Methods. Lane 1: bacteria were incubated in the isotonic component of the lambda NID extraction buffer, i.e. 5% sucrose and 50 mM Tris pH 8. 10 µl DNA were loaded in the gel. Note minimal leakage of phage particles in this lane. Lane 2: the same buffer as lane 1 was used, but with the addition of 50 mM CaCl_2_. 10 µl DNA were loaded. Note partial suppression of phage particles leakage. In lanes 3 and 4, all components of the lambda NID extraction buffer were added except lysozyme and Tween 80, respectively. 3 µl DNA were loaded. Lane 5: bacteria were incubated with the complete lambda NID extraction buffer, and 2 µl DNA were loaded. Lane 6: cells were lysed with chloroform (full lysis sample), and 0.5 µl DNA were loaded on the gel. Lanes 7 and 8: Hind III was used to digest the samples loaded in lanes 5 and 6, respectively. Densitometry analysis of the bands in lanes 1–6 is reported.

To further assess the NID procedure, we attempted to extract bacteriophage lambda particles. Lambda prophage of UB61 cells can be induced by heat shock. The phage particles accumulate in host bacteria without lysing them because of a mutation in lambda gene S. Cell lysis and release of phage particles can be achieved by briefly incubating the cells with chloroform [21]. Approximately 6 hours after heat induction of the lambda prophage and without addition of chloroform, UB-61 cells released some lambda particles in solutions of 5% sucrose and Tris (Figure 4, lane 1), which was defined as “leakage” of lambda particles. However, we found that 50 mM CaCl_2_ in the extraction buffer decreased phage leakage (Figure 4, lane 2). Both lysozyme and Tween 80 were critical for phage isolation as in the absence of either one, extraction efficiency decreased markedly compared to the buffer containing all components (Figure 4, lanes 3–5). The NID method extracted about 50% less lambda DNA compared to full cell lysis (Figure 4, lanes 5–6, see DNA loading in legend). To confirm that the bands in lanes 4 and 5 are lambda DNA, we treated both samples with HindIII to generate classical digestion products (Figure 4, lanes 7–8). These data demonstrate that NID minipreps are mild extraction procedures which can isolate various bacterial extra-chromosomal DNA elements.

### Effects of different plasmid isolation procedures on *E. coli* cells

**Figure 5:**
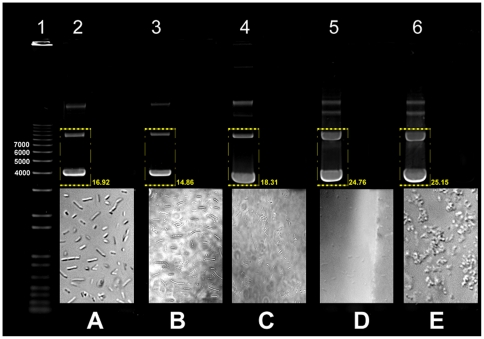
Plasmid DNA molecular forms and *E.coli* cell morphology after various isolation procedures. Plasmids were isolated from 2 ml XL1-Blue cultures containing multicopy plasmid B254, a pBluescript-based plasmid. Lane 1, 1 Kb Plus DNA ladder. Lane 2, isolation according to Godson and Sinsheimer using Brij-58. 15 µl DNA were loaded. Panel A shows corresponding *E.coli* cell morphology. Lane 3, isolation according to Clewell and Helinski using Brij-58 and DOX. In this and all following lanes 3 µl DNA were loaded. Panel B shows corresponding *E.coli* cell morphology. Lane 4, isolation according to Summerton et al. using Brij-58 and DOX in highly hypertonic salt-sucrose solutions. Panel C shows corresponding *E.coli* cell morphology. Lane 5, alkali isolation according to Birnboim and Doly. Panel D shows corresponding *E.coli* cell morphology. Lane 6, NID isolation. Panel E shows corresponding *E.coli* cell morphology. Densitometry analysis of plasmid DNA isolated with the different methods is reported.

We reasoned that the efficiency of various isolation methods might be dependent on their effects on *E. coli* cells. Thus, we assessed *E. coli* cell morphology and plasmid DNA molecular forms after the NID procedure and the following approaches: isolation using Brij-58 according to Godson and Sinsheimer [18], isolation using Brij-58 and DOX according to Clewell and Helinski [19], isolation using Brij-58 and DOX in highly hypertonic salt-sucrose solutions according to Summerton et al. [20], and the classical alkali method according to Birnboim and Doly [1]. Because the first 3 methods do not provide a miniprep protocol, we scaled-down these procedures to generate 150 µl cell lysates, matching the volume of crude extract of our NID approach. Bacteria treated with Brij-58 maintained a rod-like appearance (Figure 5, panel A), but the amounts of fast and slow-migrating plasmid DNA were low (Figure 5, lane 2). When Brij-58 and DOX were used, numerous bacteria maintained a rod-like appearance but some were disrupted (Figure 5, panel B), although the levels of fast and slow-migrating plasmid DNA remained low (Figure 5, lane 3). Isolation using Brij-58 and DOX in hypertonic salt-sucrose solutions led to a more significant degree of bacterial cell disruption (Figure 5, panel C) and higher amounts of fast-migrating plasmid DNA, but slow-migrating DNA remained low (Figure 5, lane 4). The alkali method led to complete disintegration of bacteria (Figure 5, panel D) and the levels of both fast and slow-migrating plasmid DNA were high (Figure 5, lane 5). The NID miniprep led to an intermediate degree of bacterial disruption and protoplast-like cell morphology (Figure 5, panel E) [31]. Levels of both fast and slow-migrating plasmid DNA were high (Figure 5, lane 6). To facilitate comparison of the different methods, we summarized their key features in table format (Table 2). These data reveal how different plasmid DNA isolation methods affect *E. coli* cells, and provides evidence for the connection between the bacterial cell morphology induced by a specific procedure and its efficiency.

**Table 2:** Main features of various bacterial extra-chromosomal element isolation procedures.

Methods	Detergents	Number of solutions	DNA yield	Extracted intracellular components	Miniprep procedure developed by original investigators	Insoluble component after reconstitution of DNA pellet
Godson- Sinsheimer	Brij58	3	poor	Polysomes	No	Little
Clewell-Helinski	Brij58/DOX	3	intermediate	Plasmids	No	Intermediate
Summerton et al.	Brij58/DOX	3	high	Plasmids	No	Highest
NID procedure	Only one NID with HLB≥13	1	high	Plasmids and λ phage particles	Yes	Least
Alkali procedure (Birnboim-Doly)	SDS	3	high	Plasmids	Yes	None

## Discussion

Here, we have described a one-step, one-solution inexpensive and time-efficient NID-based approach to isolate high quality plasmid DNA and lambda phage particles. This method outperforms alkali-based procedures.

To our knowledge, this is the first time miniprep crude lysates are tested in a downstream application. Our data demonstrate that NID crude lysates contain plasmid DNA that is sufficiently pure to be directly used in enzymatic reactions (Figure 3, Figure S1). It appears that crude lysates do not perform well with XhoI. However, complete NID miniprep DNA required digestion in the New England Biolabs (NEB) buffer recommended for the specific restriction endonuclease, while all NID crude lysates performed best in NEB buffer 1 (no salt), rendering the crude lysate procedure particularly convenient for most restriction endonuclease digestions. Overall, these data provide evidence that NID plasmid DNA is purer than alkali DNA and the NID miniprep is a one-step procedure (i.e., isopropanol precipitation is not required).

The phage lambda is a temperate double-stranded DNA bacteriophage harboring about 50 Kb DNA with an isometric head about 50 nm in diameter, and a flexible tail about 150 nm long. The phage linear size is approximately 10 times smaller than *E. coli* cells (1–3 µm) [32]. To release such bulky and rigid particles from infected cells *in vivo* host cell lysis is required. Products of two lambda phage genes S, and R are necessary for cell lysis. Holin, the S gene product, causes lesions (holes) in the cytoplasmic membrane at precise times of the vegetative cycle just before host lysis. The R gene product, endolysin, is a transglycosylase with murein-degrading activity. It has no secretory signal sequence and thus needs the function of S to reach the murein sacculus. Sam7, an amber mutation (Trp56UAG) of the S gene causes accumulation of intracellular virions and endolysin to very high levels. Adding chloroform, which functionally compensates holin, to an induced S^-^R^+^ lysogen (such as UB-61 cells) results in almost instantaneous lysis [33]. However, S^+^R^-^ lysogens are resistant to chloroform treatment, suggesting that some R gene product is required for chloroform-induced lysis of S^-^R^+^ lysogens [34]. We reasoned that the NIDs and lysozyme of the NID procedure might at least partially compensate for the functions of the S and R gene products. Thus, we tested a variety of NIDs, including the NIDs used in Figure 1 as well as Brij-58, Brij-98 (HLB = 15), polyoxyethylene tridecyl ether (HLB = 14) and TERGITOL TMN 10 (HLB = 14.1). However, only Tween 20, Tween 60 and Tween 80 could be used at relatively low temperatures (40°C) for phage isolation. Thus, while chloroform is only active with specific genotypes, NIDs are effective irrespectively of the phage genotype.

Current phage DNA extraction procedures usually require a purification step because DNA isolation from complete cell lysates leads to heavy contamination of phage particles with cell debris. Phenol-extracted DNA from full lysis samples not subjected to additional purification of either phage particles or phage DNA often degrades or aggregates with impurities upon storage, and it is almost completely resistant to HindIII digestion (G. L. and L.B., personal communication). However, lambda DNA isolated with phenol from NID extracted phage particles shows no degradation or aggregation after 2 month storage at 4°C (G. L. and L.B., personal communication). Thus, NID procedures do not require any specific structure, size or gene expression of extrachrosomal elements, and can simplify phage DNA purification because they do not produce complete cell lysis and NID crude lysates are relatively pure (Figure 3 and Figure S1).

We used 0.75 M salt in the extraction buffer of the NID miniprep procedure because numerous insoluble particles appeared in the DNA solution when we used 0.25 M salt (data not shown, and Table 2 for different extraction methods). However, to ensure that higher salt concentrations did not inhibit enzyme activity, we tested the DNA of the NID procedure with salt sensitive restriction endonucleases, such as SacI and KpnI (Figure 2). Previous investigations have shown that Triton X-100 (HLB 13.5) is about 3 times more effective in solubilizing *E.coli* cell membrane proteins compared to Tween 20 (HLB 16.7) [35], and high HLB NIDs do not interact easily with the plasma membrane [36]. However, Brij 58 (HLB 16) isolated plasmid DNA fairly well in conjunction with DOX (HLB 16) [19], [20], suggesting that bulky side-groups, not high HLB may be responsible for the lower efficiency of Tween 20 and Tween 80 (Figure 1). Unlike Clewell and Helinski, and Summerton et al. methods [19], [20], with our NID approach the bacterial suspension remained turbid at room temperature and even for some time after heating, indicating no significant bacterial cell lysis.

The NID miniprep uses only one solution and one tube, with no need to mix the solutions for denaturation and neutralization as it occurs in alkali minipreps, making the NID miniprep cheaper and more robust. Temperature contributed significantly to the NID miniprep because extraction increased markedly at 65°C (Figure 1, lanes 8–9). In addition, the NID miniprep DNA is a better substrate for both the restriction and ligation reactions compared to the alkali miniprep DNA (Figure 2A, lanes 5–12 and 2B). When inhibitors of DNA ligation are present, such as SDS, ligation can be incomplete, leading to monomeric or polymeric DNA forms, either liner or circular, as shown in Figure 2B, lane 2. However, complete ligation leads to one or few highly polymerized DNA molecules, as shown in Fig 2B, lane 5. Thus, we propose that SDS contamination of the alkali miniprep is likely responsible for these effects. The efficiency of different DNA isolation methods may relate to their effects on bacterial cells. With the NID miniprep, bacterial cells are not completely disrupted as in the alkali method, but appear to undergo fast re-association as protoplast-like structures which contain most of the chromosomal DNA. Overall, our data suggest that the evaluation of bacterial cell morphology is an important read-out for the efficacy of different plasmid DNA isolation methods.

NIDs are simple molecules, but their complex properties allow their use in a wide array of technological applications. In this report, we have shown that NIDs are highly effective for plasmid DNA isolation and they also extract phage lambda particles. NID minipreps with or without isopropanol precipitation (crude lysates) generate DNA of sufficiently high quality for common downstream applications. The timed saved by the NID miniprep increases rapidly at higher sample numbers, suggesting that NID minipreps are particularly suitable for high-throughput applications. Overall, the NID miniprep outperforms current alkali plasmid DNA isolation methods, and represents a new standard in plasmid DNA minipreps.

## Supporting Information

Figure S1 **NID crude lysates can be used in sequencing reactions.** NID crude lysates of XL1-Blue cells harboring B256 plasmid were prepared as described in Materials and Methods but using 5 mM EDTA in the extraction buffer. Lysate EDTA was chelated with 5 mM MgSO_4_ before the sequencing reaction. 12 µl primer/crude lysate mix containing 4 pmoles M13f primer was diluted by adding 15 µl water. 6 µl of this diluted mix and 4 µl of Big Dye mix made up the sequencing reaction mixes. (**A**): 1 µl crude lysate. (**B**): 3 µl crude lysates. (**C**): 4 µl crude lysates.
(TIF)
