# Supplementary figures and images for: A One-Step Miniprep for the Isolation of Plasmid DNA and Lambda Phage Particles
Source: PLoS One. 2011 Aug 15;6(8):e23457. doi: 10.1371/journal.pone.0023457 (PMC3156146; doi:10.1371/journal.pone.0023457)

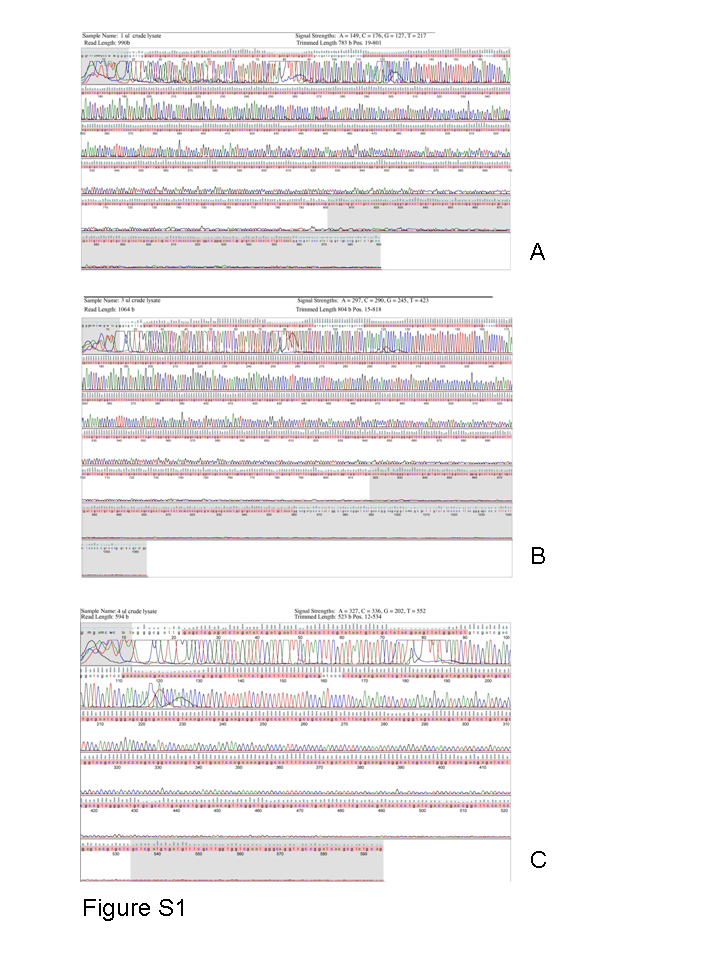

Supplement: Figure S1 — NID crude lysates can be used in sequencing reactions. NID crude lysates of XL1-Blue cells harboring B256 plasmid were prepared as described in Materials and Methods but using 5 mM EDTA in the extraction buffer. Lysate EDTA was chelated with 5 mM MgSO4 before the sequencing reaction. 12 µl primer/crude lysate mix containing 4 pmoles M13f primer was diluted by adding 15 µl water. 6 µl of this diluted mix and 4 µl of Big Dye mix made up the sequencing reaction mixes. (A): 1 µl crude lysate. (B): 3 µl crude lysates. (C): 4 µl crude lysates. (TIF) [file pone.0023457.s001.tif]
